# Working situation and burden of work limitations in sarcoma patients: results from the multi-center prospective PROSa study

**DOI:** 10.1007/s00432-022-04556-3

**Published:** 2023-01-10

**Authors:** Sergio Armando Zapata Bonilla, Marius Fried, Susanne Singer, Leopold Hentschel, Stephan Richter, Peter Hohenberger, Bernd Kasper, Dimosthenis Andreou, Daniel Pink, Karin Arndt, Martin Bornhäuser, Jochen Schmitt, Markus K. Schuler, Martin Eichler

**Affiliations:** 1 Clinic and Polyclinic for Internal Medicine III, Haematology and Medical Oncology/University Centre for Tumor Diseases (UCT), University Hospital Johannes Gutenberg, University Hospital Mainz, Mainz, Germany; 2 Institute for Medical Biostatistics, Epidemiology and Informatics, University Hospital Mainz, Mainz, Germany; 3 National Center for Tumor Diseases Dresden (NCT/UCC), Dresden, Germany; 4 German Cancer Research Center (DKFZ), Heidelberg, Germany; 5 Faculty of Medicine and University Hospital Carl Gustav Carus, Technical University Dresden, Dresden, Germany; 6 Helmholtz-Zentrum Dresden-Rossendorf (HZDR), Dresden, Germany; 7 Clinic and Polyclinic for Internal Medicine I, University Hospital Carl Gustav Carus, Dresden, Germany; 8 Division of Surgical Oncology & Thoracic Surgery, Mannheim University Medical Center, University of Heidelberg, Heidelberg, Germany; 9 Sarcoma Unit, Mannheim Cancer Center (MCC), Mannheim University Medical Center, University of Heidelberg, Mannheim, Germany; 10 Department of General Orthopedics and Tumor Orthopedics, University Hospital Munster, Munster, Germany; 11 Department of Orthopedics and Trauma, Medical University of Graz, Graz, Austria; 12 Sarcoma Center Berlin-Brandenburg, Helios Hospital Bad Saarow, Brandenburg, Germany; 13 Department of Internal Medicine C, University Hospital Greifswald, Greifswald, Germany; 14 German Sarcoma Foundation, Woelfersheim, Germany; 15 Center for Evidence-Based Healthcare, Medizinische Fakultät Carl Gustav Carus, TU, Dresden, Germany

**Keywords:** Sarcoma, Rare diseases, Working situation, Limitations at work, Disability pension, Drop out of work, Return to work, Work-limitations questionnaire

## Abstract

**Purpose:**

We investigated predictors of limitations in work performance, odds of drop out of work, and odds of receiving disability pension in sarcoma patients.

**Methods:**

We measured clinical and sociodemographic data in adult sarcoma patients and recorded if the patients received a (1) disability pension at baseline or (2) had dropped out of work 1 year after initial assessment. (3) Work limitations were assessed using the Work-limitations questionnaire (WLQ^©^). We analyzed exploratively.

**Results:**

(1) Amongst 364 analyzed patients, odds to receive a disability pension were higher in patients with abdominal tumors, older patients, high grade patients and with increasing time since diagnosis. (2) Of 356 patients employed at baseline, 21% (*n* = 76) had dropped out of work after 1 year. The odds of dropping out of work were higher in bone sarcoma patients and in patients who received additive radiotherapy ± systemic therapy compared with patients who received surgery alone. Odds of dropping out of work were less amongst self-employed patients and dropped with increasing time since diagnosis. (3) Work limitations were higher in woman and increased with age. Patients with bone and fibrous sarcomas were more affected than liposarcoma patients. Patients with abdominal tumors reported highest restrictions. Sarcoma treatment in the last 6 months increased work limitations.

**Conclusion:**

Work limitations, drop out of work and dependence on a disability pension occurs frequently in patients with sarcoma adding to the burden of this condition. We were able to identify vulnerable groups in both the socioeconomic and disease categories.

**Supplementary Information:**

The online version contains supplementary material available at 10.1007/s00432-022-04556-3.

## Introduction

An increasing number of papers report on issues concerning employment and work of patients surviving cancer. Given the increasing number of cancer survivors, which is reported to have risen by 60%, their ability to return and remain employed is also of social relevance. Around 40% of cancer survivors are under 65 years of age (Vecchia et al. 2015; Butow et al. 2020) and thus under a favorable health status may return to the workforce. In Germany, for example, there are currently 3.1 million cancer survivors (Arndt et al. 2021). The average rate of return to work amongst cancer survivors has been reported to be around 60%, however, with a wide range from 24 to 94% depending on the malignancy (Mehnert 2011). The return-to-work rate of prostate cancer patients was 80% (McLennan et al. 2019), while for hematological malignancies this was only 58% (Hartung et al. 2018) and for colorectal cancers even 37% (Bakker et al. 2020). In general, people with cancer are slightly less often employed than people without cancer, mainly because of their higher retirement rate (34% vs. 27%). The employment rate of the people with cancer varies greatly according to the cancer site with early retirement being more common among people with highly disabling cancer or poor prognosis (Taskila-Abrandt et al. 2005).

Oftentimes patients instead of re-entering the workforce opt for early retirement or disability pension after surviving cancer. In Germany anyone who due to illness or accident cannot or only partially work, may receive a disability pension. In 2020, this amounted to 1.82 Million people of whom 20% were below 50 years of age (Deutsche Rentenversicherung Bund 2021) and cancer patients have an increase relative risk of receiving an early retirement pension. A population-wide study also reported an increased relative risk of an early retirement of cancer patients in Denmark (Carlsen et al. 2008).

Return to work (RTW) is currently the most investigated work-related research question. Returning to and staying in the job is for many patients not only of social benefit but also important for their identity, societal role, life-purpose and a return to normality, all of which are determinants of wellbeing and for quality of life. Nonetheless, the ability to work is not a given for many cancer survivors. Many of them suffer from a variety of physical, social, and mental health problems, sometimes as a sequel of the therapies. Strong evidence suggests that physical exertion, type of surgery, chemotherapy, cancer site, and type of malignancy are prognostic factors for RTW or non RTW (Horsboel et al. 2013; Muijen et al. 2013). In addition, socioeconomic factors, such as education, income and type of work, are predictive factors for a successful RTW, in particular for survivors of cancers in the musculoskeletal system, e.g. sarcoma patients (Cancelliere et al. 2016).

So far only few studies have included subjective measures of work ability or limitations, and those who have, have mostly focused on perceived work ability, self-efficacy and/or fatigue after RTW (Wolvers et al. 2018; Muijen et al. 2017).

With this paper, we focus on the inability to work and work limitations of sarcoma patients and survivors. Here too, an increase in survival is making RTW an increasingly important topic for patients (Blay et al. 2019), especially as the disease affects often people of working age (Stiller et al. 2013). To our knowledge, studies exploring work-related topics in sarcoma patients and survivors are scarce and focus mostly on patients with sarcoma in just one localization, namely the extremities (Zambrano et al. 2020; Parsons et al. 2008; Kwong et al. 2014; Kollár et al. 2021). For these the rate of RTW has been reported at 89% (Kollár et al. 2021). However, sarcomas are a heterogeneous group of tumors with > 100 histological subtypes (Fletcher et al. 2013), affect a range of body areas, and its therapy is complex and with divergent treatment algorithms (Casali et al. 2018)—all of which can result in physical disabilities (Parsons et al. 2008). Consequently it is to be expected that work related abilities and disabilities differ among sarcoma patient subgroups especially with regard to localization and type of sarcoma but different more or less aggressive treatment modalities probably play a role as well.

We investigated the following research questions:

1. What factors are associated with the odds to become dependent on disability pension during disease course?
2. What factors at the time of study enrollment (baseline) are associated with sarcoma patients having to drop out of work in the course of one year?
3. What are predictors of limitations at the work place amongst this working sarcoma patients and survivors?

## Patients and methods

**Fig. 1:**
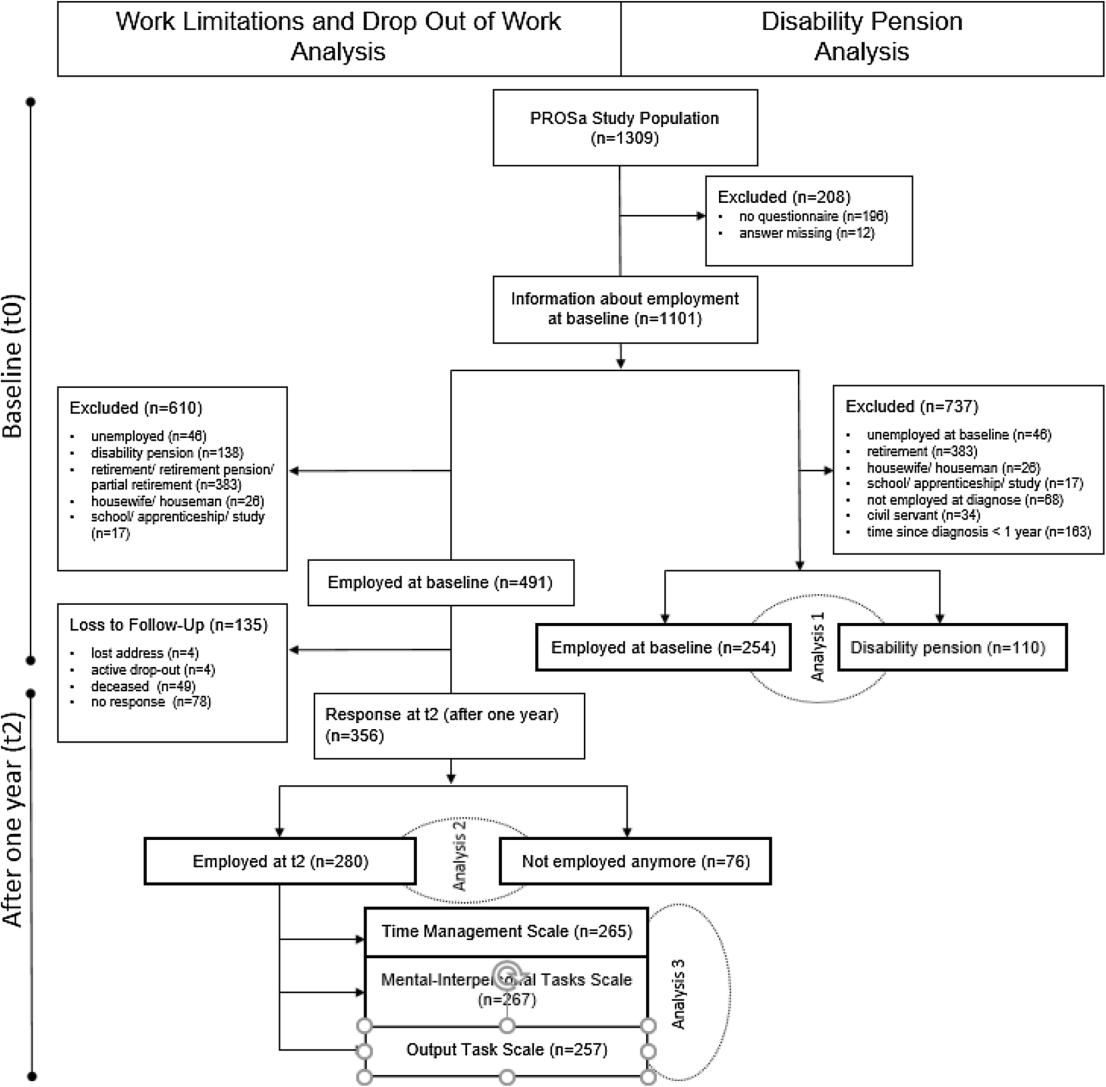
Flow chart study population

The prospective PROSa cohort study (Burden and Medical Care of Sarcoma in Germany: Nationwide Cohort Study Focusing on Modifiable Determinants of Patient-Reported Outcome Measures in Sarcoma Patients) (www.uniklinikum-dresden.de/prosastudie) with a one-year follow-up time (t2) was conducted nationwide from September 2017 to May 2020 in 39 study centers in Germany (NCT03521531; ClinicalTrials.gov). PROSa gathered information on a range of clinical data (e.g., sex, age at diagnosis, type of sarcoma, localization, type of treatment, malignancy grading and tumor size), socioeconomic factors (i.e. type of education and type of occupation at study entry), and patient reported outcomes of patients with prevalent sarcoma. Data were collected at baseline (t0), 6 (t1) and 12 (t2) months after study inclusion. Here, we analyzed data of adult patients with histologically confirmed proven sarcoma of any entity. Patients who were mentally or linguistically unable to complete questionnaires and those with missing data on employment status were excluded. For research question 1, we analyzed patients who were employed or self-employed at time of diagnosis and excluded patients who were at baseline unemployed, retired, housewife/houseman or at school, in an apprenticeship or in study. As the entitlement to get a disability pension is subject to certain formal preconditions, we excluded civil servants and those patients who were diagnosed less than a year ago. For research question 2, we analyzed only patients working at baseline and with data on employment status at t2. For research question 3, we analyzed only participants with completed questionnaires at t2 (see Fig. 1). Eligible patients were asked to participate at the referral centers during visits, and at times by phone or letter. Participation required written informed consent. The study was advised and approved by the ethics committees of the Technical University of Dresden (EK1790422017) and the participating centers. Completed questionnaires were sent by the participants to the study center by mail or online. Clinical information was submitted online by the participating centers using documentation forms. Data collection was performed using REDCap (Harris et al. 2009). More detailed information on study design and participation are available (Eichler et al. 2021).

### Outcomes and variables

To explore possible factors associated with receiving a disability pension (research question 1), we examined sex (female, male), age at study entry (18– < 40, 40– < 55, > 55 years), and the following socioeconomic variables: school education (secondary school (8 or 9 years), secondary school (10 years), baccalaureate, other), occupation (blue collar worker, white collar worker, self-employed, other/ unknown), As clinical factors, we analyzed time since diagnosis (1–2 years, 2–5 years, ≥ 5 years), sarcoma type (liposarcoma, bone sarcoma, GIST, unclassified, fibroblastic/myofibroblastic/fibrohistiocytic sarcoma, leiomyosarcoma, other soft tissue sarcoma), tumor location (abdomen + retroperitoneum, thorax, pelvis, lower limbs, upper limbs, other), histological grading (low, high, unknown).

To investigate possible factors associated with dropping out of work (research question 2), we examined the variables mentioned above. Additionally, we included civil servants as category in occupation and 0–0.5 year and 0.5– < 1 year as categories in time since diagnosis. As treatment related factors, we analyzed: treatment at baseline (in treatment, not in treatment), disease status at baseline (complete response, partial response/stable disease, progressive disease, unknown) and type of received treatment until t0 (surgery only, surgery + systemic therapy, surgery + radiotherapy, surgery + systemic therapy + radiotherapy, none, other).

To evaluate possible factors associated with limitations at the work place (research question 3) we examined the variables mentioned above, but made changes with regard of the treatment associated factors. Here, we analyzed if the patient received any kind of treatment in the last 6 months until t2 (instead of treatment at t0).

For research questions 3 we used the scales from the Work Limitation Questionnaire Version 1.0 (WLQ) (Williams 2001; Arumugam and MacDermid 2013) as outcome variables. This instrument has 25 items which are aggregated into four different scales: time management scale, physical tasks scale, mental-interpersonal tasks scale, and output tasks scale. The score for each scale ranges from 0 (limited none of the time) to 100 (limited all of the time). We used the scales: (1) Time Management (difficulties handling time and scheduling demands), (2) Mental-Interpersonal-Tasks (cognitive tasks and social interactions at work), and (3) Output Tasks (diminished work quantity and quality).

### Statistical analysis

When normally distributed, continuous variables were presented with mean and standard deviation (SD), with median and interquartile range if this was not the case. Categorical variables were reported with absolute and relative frequencies.

For research question 1 and 2, multivariable binary logistic regressions without variable selection were fitted. For research question 3, we used Generalized Linear Regression models without variable selection. In all cases confidence intervals with 95% were calculated. Statistical analyses were exploratory and performed using SPSS V.26 (IBM Corporation, Armonk, New York, USA). The categorical, independent variables with more than 3 missing values were compiled in the variable “unknown”, otherwise cases were excluded.

## Results

As shown in Fig. 1, the study population of PROSa study consisted of 1309 patients. Information about employment at baseline were available from 1101 patients. For research question 1, we analyzed 364 patients, who were either employed (*n* = 254) or received a disability pension at baseline (*n* = 110), thereby excluding those not entitled to receive this kind of social welfare. For research question 2, we analyzed 356 patients (*n* = 280 in employment, *n* = 76 not in employment) at t2 we had been employed at baseline 2. We lost 135 patients out of a total 491 during follow up.

### Descriptive characteristics study population

The table showing the characteristics of the population analyzed for research question 1 can be found in the online appendix. Among the population at risk to drop out of work at t2 (research questions 2) were 173 (49%) female and 183 (51%) male; the age distribution was as follows, 88 (25%) were 18–39, 140 (39%) were 40–54, and 128 (36%) were > 55 years (Table 1). Almost half of the patients (*n* = 176; 49.4%) had a high school or baccalaureate education and more than half (*n* = 220; 61.8%) were white collar workers. The most represented Sarcoma subtype was liposarcoma (*n* = 74; 20.8%) and the most common localization were the lower limbs (*n* = 147; 41.4%). Half of the sarcomas were classified as high grade (*n* = 183; 51.4%). The vast majority of the patients (*n* = 269; 75.6%) were not in treatment at t0.

**Table 1:** Drop out of work during follow up

Variable-value	Not in employment anymore *N* = 76 (21.3%) *N* (%)	In employment at t2 *N* = 280 (77.7%) *N* (%)	All *N* = 356 *N* (%)
Sex			
Female	37 (21.4)	136 (78.6)	173 (48.6)
Male	39 (21.3)	144 (78.7)	183 (51.4)
Age at study inclusion			
18– < 40 years	18 (20.5)	70 (79.5)	88 (24.7)
40– < 55 years	27 (19.3)	113 (80.7)	140 (39.3)
55 years and older	31 (24.2)	97 (75.8)	128 (36.0)
School education			
Secondary school (8/9 years)	10 (22.2)	35 (77.8)	45 (12.7)
Secondary school (10 years)	35 (26.7)	96 (73.3)	131 (36.8)
(Vocational) baccalaureate/high school	31 (17.6)	145 (82.4)	176 (49.4)
Other	0 (0.0)	4 (100.0)	4 (1.1)
Occupational status			
Blue collar worker	22 (32.4)	46 (67.6)	68 (19.1)
Civil servant	3 (11.5)	23 (88.5)	26 (7.3)
White collar worker	49 (22.3)	171 (77.7)	220 (61.8)
Self employed	2 (6.1)	31 (93.9)	33 (9.3)
Not applicable/unknown	0 (0.0)	9 (100.0)	9 (2.5)
Time since diagnosis t0			
0– < 0.5 year	30 (37.5)	50 (62.5)	80 (22.5)
0.5– < 1 year	12 (26.7)	33 (73.3)	45 (12.6)
1– < 2 years	14 (26.4)	39 (73.6)	53 (14.9)
2– < 5 years	10 (11.8)	75 (88.2)	85 (23.9)
> 5 years	10 (10.8)	83 (89.2)	93 (26.1)
Sarcoma type			
Liposarcoma	9 (12.2)	65 (87.8)	74 (20.8)
Bone sarcoma	23 (26.4)	64 (73.6)	87 (24.4)
GIST	7 (21.2)	26 (78.8)	33 (9.3)
fibroblastic, myofibroblastic, fibrohistiocytic sarcoma	3 (6.7)	42 (93.3)	45 (12.6)
Unclassified sarcoma	9 (21.4)	33 (78.6)	42 (11.8)
Leiomyosarcoma	13 (37.1)	22 (62.9)	35 (9.8)
Other soft tissue sarcoma	12 (30.0)	28 (70.0)	40 (11.2)
Site			
Abdomen/retroperitoneum	14 (19.2)	59 (80.8)	73 (20.5)
Thorax	7 (21.2)	26 (78.8)	33 (9.3)
Pelvis	18 (32.1)	38 (67.9)	56 (15.7)
Lower limbs	24 (16.3)	123 (83.7)	147 (41.4)
Upper limbs	8 (27.6)	21 (72.4)	29 (8.1)
Other	5 (27.8)	13 (72.2)	18 (5.0)
Grading at diagnose			
Low grade	8 (13.8)	50 (86.2)	58 (16.3)
High grade	48 (26.2)	135 (73.8)	183 (51.4)
Not applicable	17 (17.3)	81 (82.7)	98 (27.5)
Unknown	3 (17.6)	14 (82.4)	17 (4.8)
Treatment status at baseline			
In treatment	31 (35.6)	56 (64.4)	87 (24.4)
Not in treatment	45 (16.7)	224 (83.3)	269 (75.6)
Disease status at baseline			
Complete remission	36 (18.8)	155 (81.2)	191 (53.7)
Partial remission/stable disease	12 (12.5)	84 (87.5)	96 (27.0)
Progress	11(45.8)	13 (54.2)	24 (6.7)
Unknown/not accessible	17 (37.8)	28 (62.2)	45 (12.6)
Received Treatments until baseline			
Surgery only	18 (14.2)	109 (85.5)	127 (35.6)
Surgery + systemic therapy	17 (23.3)	56 (76.7)	73 (20.5)
Surgery + radiotherapy	10 (16.9)	49 (83.1)	59 (16.6)
Surgery + systemic therapy + radiotherapy	16 (27.1)	43 (72.9)	59 (16.6)
None at t0	5 (33.3)	10 (66.7)	15 (4.2)
Other	10 (43.5)	13 (56.5)	23 (6.5)

Frequencies

*GIST* gastrointestinal stromal tumor

### Analysis 1–receiving a disability pension—associated factors

Odds to receive a disability pension over disease course increased in the higher age groups (40– < 55 years: OR 3.5; 95% CI 1.4–8.8; ≥ 55 years: OR 4.3; 95% CI 1.6–11.3) (Table 2). Self-employed patients had lesser odds (OR 0.15; 95% CI 0.04–0.58) compared with other occupational groups. Increasing time with diagnosis was associated with higher odds of receiving a disability pension (2– < 5 years: OR 2.64; 95% CI 1.19–5.83, > 5 years: OR 3.63; 95% CI 1.64–8.03). Patients with a diagnosis of “other soft tissue sarcoma” had also higher odds of receiving a disability pension compared with other histological types (OR 2.89; 95% CI 1.03–8.11). Also, odds were higher in patients with retroperitoneal/ abdominal tumors compared to patients with thoracic (OR 0.18; 95% CI 0.05–0.65 or extremity tumors (lower limbs: OR 0.31; 95% CI 0.13–0.72; upper limbs: OR 0.13; 95% CI 0.03–0.58) and in high grade patients (OR 3.5; 95% CI 1.38–8.90).

**Table 2:** Disability pension at baseline

Variable-value	Not in employment anymore OR (95% CI), *p* =
Sex—male vs. female	0.63 (0.35–1.12), *p* = 0.11
Age at study inclusion	
18– < 40 years	Ref
40– < 55 years	**3.45 (1.35**–**8.84), *p* = 0.01**
≥ 55 years	**4.29 (1.63**–**11.31), *p* < 0.01**
School education	
Secondary school (8/9 years)	Ref
Secondary school (10 years)	1.13 (0.53–2.43), *p* = 0.75
(Vocational) baccalaureate/high school	0.45 (0.19–1.07), *p* = 0.07
Occupational status	
Blue collar worker	Ref
White collar worker	0.58 (0.27–1.24), *p* = 0.16
Self employed	**0.15 (0.04**–**0.58), *p* < 0.01**
Time since diagnosis	
1– < 2 years	Ref
2– < 5 years	**2.64 (1.19**–**5.83), *p* = 0.02**
> 5 years	**3.63 (1.64**–**8.03), *p* < 0.01**
Sarcoma type	
Liposarcoma	Ref
Bone sarcoma	1.39 (0.50–3.92), *p* = 0.53
GIST	0.99 (0.29–3.36), *p* = 0.98
Unclassified sarcoma	1.49 (0.46–4.77), *p* = 0.51
Fibroblastic, myofibroblastic, fibrohistiocytic sarcoma	1.21 (0.43–3.44), *p* = 0.72
Leiomyosarcoma	1.94 (0.72–5.23**)**, *p* = 0.19
Other soft tissue sarcoma	**2.89 (1.03**–**8.11), *p* = 0.04**
Site	
Abdomen/retroperitoneum	Ref
Thorax	**0.18 (0.05**–**0.65), *p* < 0.01**
Pelvis	0.47 (0.18–1.23), *p* = 0.13
Lower limbs	**0.31 (0.13**–**0.72), *p* < 0.01**
Upper limbs	**0.13 (0.03**–**0.58), *p* < 0.01**
Other	1.52 (0.40–5.74), *p* = 0.54
Grading at diagnose	
Low grade	Ref
High grade	**3.50 (1.38**–**8.90), *p* < 0.01**
Not applicable	1.49 (0.49–4.60), *p* = 0.48
Unknown	**3.90 (1.15**–**13.20), *p* = 0.03**

Multivariate logistic regression

Significant results: bold

*GIST* gastrointestinal stromal tumor, *OR* odds ratio, *p* p value

### Analysis 2–drop out of work—associated factors

Self-employed patients were less likely to drop out work one after one year (OR 0.06; 95% CI 0.01–0.37) (Table 3). Time since diagnosis was independently associated with having dropped out of work: if the sarcoma was diagnosed more than two years prior to enrollment, the patients were less likely to drop out of work (2– < 5 years: OR 0.16; 95% CI 0.05–0.53, > 5 years: OR 0.16; 95% CI 0.05–0.53). Regarding the histological type, bone sarcomas (OR 5.33; 95% CI 1.67–17.14) and other soft tissue sarcomas (OR 3.87; 95% CI 1.13–13.2) were more likely to stop working compared with liposarcoma patients. Patients with partial remission or stable disease dropped out of work significantly less often than patients in complete remission (OR 0.27; 95% CI 0.11–0.68). Regarding type of therapy, patients who had received a combined therapy including surgery plus (systemic) plus radiotherapy were more likely to have dropped out of work after one year (surgery + radiotherapy: OR 3.29; 95% CI 1.11–9.77, surgery + systemic + radiotherapy: OR 4.13; 95% CI 1.46–11.69).

**Table 3:** Drop out of work at t2

Variable-value	Not in employment anymore OR (95% CI), *p*
Sex—male vs. female	0.86 (0.43–1.74), *p* = 0.68
Age at study inclusion	
18– < 40 years	Ref
40– < 55 years	0.92 (0.3–2.22), *p* = 0.84
> 55 years	2.25 (0.90–5.62), *p* = 0.08
School education	
Secondary school (8/9 years)	Ref
Secondary school (10 years)	2.41 (0.81–7.16), *p* = 0.11
(Vocational) baccalaureate/high school	1.49 (0.46–4.84), *p* = 0.51
Occupational status	
Blue collar worker	Ref
Civil servant	0.22 (0.04–1.06), *p* = 0.06
White collar worker	0.46 (0.19–1.11), *p* = 0.08
Self employed	**0.06 (0.01**–**0.37), ** ***p* = < 0.01**
Time since diagnosis at baseline	
0– < 0.5 year	Ref
0.5– < 1 year	0.43 (0.17–1.62), *p* = 0.26
1– < 2 years	0.58 (0.19–1.79), *p* = 0.34
2– < 5 years	**0.16 (0.05**–**0.53), ** ***p* = < 0.01**
> 5 years	**0.16 (0.05**–**0.53), ** ***p* = < 0.01**
Sarcoma type	
Liposarcoma	Ref
Bone sarcoma	**5.33 (1.67**–**17.14), ** ***p* < 0.01**
GIST	5.59 (0.88–35.64), *p* = 0.07
Fibroblastic, myofibroblastic, fibrohistiocytic Sarcoma	1.45 (0.42–5.02), *p* = 0.56
Unclassified sarcoma	0.75 (0.15–3.67), *p* = 0.72
Leiomyosarcoma	2.93 (0.88–9.82), *p* = 0.08
Other soft tissue sarcoma	**3.87 (1.13**–**13.2), ** ***p* = 0.03**
Site	
Abdomen/retroperitoneum	Ref
Thorax	0.49 (0.10–2.35), *p* = 0.38
Pelvis	1.54 (0.42–5.59), *p* = 0.52
Lower limbs	0.52 (0.15–1.78), *p* = 0.30
Upper limbs	0.70 (0.15–3.19), *p* = 0.65
Other	0.71 (0.12–4.12), *p* = 0.70
Grading at diagnose	
Low grade	Ref
High grade	1.59 (0.57–4.47), *p* = 0.38
Not applicable	0.94 (0.26–3.40), *p* = 0.93
Unknown	1.45 (0.23–7.86), *p* = 0.75
Treatment status at t0 (no vs. yes)	2.14 (0.81–5.65), *p* = 0.13
Disease status at t0	
Complete remission	Ref
Partial remission/stable disease	**0.27 (0.11**–**0.68), ** ***p* = < 0.01**
Progress	2.35 (0.73–7.59), *p* = 0.16
Unknown/not accessible	0.67 (0.21–2.13), *p* = 0.51
Received treatments until t0	
Surgery only	Ref
Surgery + systemic therapy	1.02 (0.36–2.85), *p* = 0.98
Surgery + radiotherapy	**3.29 (1.11**–**9.77), ** ***p* = 0.03**
Surgery + systemic therapy + radiotherapy	**4.13 (1.46**–**11.69), ** ***p* = < 0.01**
None at t0	1.74 (0.33–9.11), *p* = 0.51
Other	2.13 (0.57–7.96), *p* = 0.27

Multivariate analysis

Significant results: bold

*GIST* gastrointestinal stromal tumor, *OR* odds ratio, *p* p value

### Analysis 3–predictors of limitations at the work place amongst patients who were still working after 1 year—associated factors

For research question 3 we were able to analyze the data from 257 to 267 Patients, depending on the analyzed scale (Table 4).

**Table 4:** Factors associated with limitations at the work place in sarcoma patients

Variable-value	Scale 1—time management (N = 265) B (95% CI), *p*	Scale 2—mental-interpersonal tasks (*N* = 267) B (95% CI), *p*	Scale 3—output tasks (*N* = 257) B (95% CI), *p*
Sex			
Female	Ref	Ref	Ref
Male	− **8.1 (− 14.1 to **− **2.0), ** ***p* < 0.01**	− 4.4 (− 9.3 to 0.5), *p* = 0.08	− **6.8 (**− **12.7 to **− **1.0), ** ***p* = 0.02**
Age at study inclusion			
18– < 40 years	Ref	Ref	Ref
40– < 55 years	**11.3 (3.7 to 19.0), ** ***p* < 0.01**	5.1 (− 1.2 to 11.2), *p* = 0.11	**10.1 (2.7 to 17.5), ** ***p* < 0.01**
55 years and older	**10.6 (2.1 to 19.1), ** ***p* = 0.02**	4.7 (2.3 to 11.7), *p* = 0.19	**9.0 (0.6 to 17.3), ** ***p* = 0.04**
School education			
Secondary school (8/9 years)	Ref	Ref	Ref
Secondary school (10 years)	− 5.3 (− 15.2 to 4.5), *p* = 0.29	− 5.0 (− 13.1 to 3.0), *p* = 0.22	− 7.1 (− 16.9 to 2.6), *p* = 0.15
(Vocational) baccalaureate/high school	− 4.3 (− 14.3 to 5.7), *p* = 0.40	− 4.5 (− 12.7 to 3.6), *p* = 0.28	− 8.6 (− 18.6 to 1.3), *p* = 0.09
Occupational status			
Blue collar worker	Ref	Ref	Ref
Civil servant	− 9.7 (− 22.2 to 2.8), *p* = 0.12	− 3.4 − 13.6 to 6.9), *p* = 0.52	− 4.7 (− 17.0 to 7.4), *p* = 0.44
White collar worker	− **9.2 (**− **17.6** to − **0.7), ** ***p* = 0.04**	− 4.0 (− 11.0 to 3.0), *p* = 0.21	− 3.2 (− 11.6 to 5.2), *p* = 0.45
Self employed	− 8.1 (− 19.5 to 3.4), *p* = 0.17	− 3.9 (− 13.3 to 5.3), *p* = 0.41	− 0.7 (− 11.9 to 10.5), *p* = 0.91
Time since diagnosis			
1– < 2 years	Ref	Ref	Ref
2– < 3 years	0.5 (− 9.0 to 10.0), *p* = 0.92	1.4 (− 6.2 to 9.1), *p* = 0.71	− 1.3 (− 10.3 to 7.7), *p* = 0.78
3– < 5 years	− 5.6 (− 13.4 to 2.2), *p* = 0.16	− 3.8 (− 10.2 to 2.6), *p* = 0.24	− 5.9 (− 13.6 to 1.8), *p* = 0.13
More than 5 years	− 4.5 (− 12.1 to 3.0), *p* = 0.24	− 3.9 (− 9.9 to 2.2), *p* = 0.21	− 5.8 (− 13.1 to 1.5), *p* = 0.12
Sarcoma type			
Liposarcoma	Ref	Ref	Ref
Bone sarcoma	**15.5 (**− **4.9** to **26.2), *p* < 0.01**	**10.0 (1.3** to **18.7), *p* = 0.02**	8.2 (− 2.0 to 18.5), *p* = 0.12
GIST	2.9 (− 18.0 to 12.1), *p* = 0.70	− 2.6 (− 14.8 to 9.6), *p* = 0.68	− 9.1 (− 23.8 to 5.5), *p* = 0.22
Fibroblastic, myofibroblastic, fibrohistiocytic sarcoma	8.0 (− 2.7 to 18.6), *p* = 0.14	**9.9 (1.3** to **18.4), *p* = 0.02**	**11.2 (1.0** to **21.4), *p* = 0.03**
Unclassified sarcoma	1.2 (− 9.2 to 11.7), *p* = 0.82	4.0 (4.5 to 12.4), *p* = 0.36	− 0.5 (− 10.6 to 9.6), *p* = 0.92
Leiomyosarcoma	− 4.3 (− 15.8 to 7.7), *p* = 0.50	− 1.2 (− 10.8 to 8.3), *p* = 0.80	− 3.0 (− 14.6 to 8.6), *p* = 0.61
Other soft tissue sarcoma	**12.6 (1.1** to **24.1), *p* = 0.03**	**13.4 (4.0** to **22.8), *p* > 0.01**	**13.2 (2.0** to **24.4), *p* = 0.02**
Site			
Abdomen/retroperitoneum	Ref	Ref	Ref
Thorax	− 12.4 (− 25.6 to 0.8), *p* = 0.07	− 6.2 (− 16.9 to 4.5), *p* = 0.26	− 10.0 (− 23.0 to 3.0), *p* = 0.13
Pelvis	− 10.4 (− 22.0 to 1.1), *p* = 0.08	− 4.4 (− 13.7 to 5.0), *p* = 0.36	− 7.4 (− 18.6 to 3.7), *p* = 0.19
Lower limbs	− **11.4 (**− **21.6** to − **1.2), *p* = 0.03**	− 7.6 (− 15.8 to 0.6), *p* = 0.07	− **10.0 (-19.7** to − **0.3) **,***p* = 0.04**
Upper limbs	− **21.4 (**− **36.0** to **-6.8), *p* < 0.01**	− **16.4 (**− **28.2** to − **4.6), *p* < 0.01**	− **19.9 (**− **34.0** to − **5.8) **,***p* < 0.01**
Other	− 10.9 − 27.7 to 5.9), *p* = 0.20	− 8.0 (− 21.0 to 5.0), *p* = 0.23	− 14.8 (− 30.1 to 0.5), *p* = 0.06
Grading at diagnose			
Low grade	Ref	Ref	Ref
High grade	3.7 (− 4.7 to 12.2), *p* = 0.39	2.2 (− 4.7 to 9.0), *p* = 0.54	3.1 (− 5.1 to 11.4), *p* = 0.45
Not applicable	− 0.7 (− 10.7 to 9.3), *p* = 0.89	− 1.9 (− 10.1 to 6.3), *p* = 0.65	− 0.3 (− 9.3 to 10.0), *p* = 0.95
Unknown	9.0 (− 5.3 to 23.3), *p* = 0.22	8.5 (− 3.2 to 20.3), *p* = 0.15	2.4 (− 11.5 to 16.2), *p* = 0.74
Treated in the last 6 months at t2			
No treatment last 6 months	Ref	Ref	Ref
Treatment in the last 6 months	**19.2 (9.4 to 29.0), *p* < 0.001**	**15.4 (7.4 to 23.4), *p* < 0.001**	**14.9 (5.2 to 24.6), *p* < 0.01**
No data available	− 1.1 (− 7.8 to 5.6), *p* = 0.74	0.4 (− 5.1 to 5.9), *p* = 0.89	1.6 (− 4.9 to 8.1), *p* = 0.64
Received treatments until t2			
Surgery only	Ref	Ref	Ref
Surgery + system. Therapy	− 0.7 (− 9.2 to 7.7), *p* = 0.86	− 1.1 (− 8.0 to 5.8), *p* = 0.75	1.5 (− 6.8 to 9.8), *p* = 0.72
Surgery + radiotherapy	5.1 (− 3.4 to 13.7), *p* = 0.24	− 0.5 (− 7.4 to 6.4), *p* = 0.89	1.1 (− 7.2 to 9.4), *p* = 0.80
Surgery + system. Therapy + radiotherapy	3.4 (− 5.8 to 12.7), *p* = 0.46	1.6 (− 5.9 to 9.1), *p* = 0.67	4.6 (− 4.4 to 13.6), *p* = 0.32
Other	4.4 (− 15.2 to 24.0), *p* = 0.66	8.7 (− 7.3 to 24.7), *p* = 0.29	3.2 (− 15.6 to 22.0), *p* = 0.74

Multivariate analysis

Significant results: bold

*GIST* gastrointestinal stromal tumor, *B* unstandardized regression coefficient, *p* p value

#### Time management

Men were significantly less affected by time management problems than women (*B* = − 8.1; 95% CI − 14.1 to − 2.0). Patients > 40 years were significantly more affected than younger patients (40– < 55 years: *B* = 11.3; 95% CI 3.7–19.0, > 55 years: *B* = 10.6; 95% CI 2.1–19.1). Regarding occupational status, white collar workers were significantly less affected than other types of workers (B = − 9.2; 95% CI − 17.6 to − 0.7). Patients with a diagnosis of bone sarcoma (*B* = 15.5; 95% CI − 4.9 to 26.2) and other types of sarcomas (*B* = 12.6; 95% CI 1.1–24.1) were also more limited in their time management in comparison to liposarcoma. Abdominal and/or retroperitoneal sarcomas were the most burdened, using them as reference all other locations were less affected, significantly the upper and lower extremities (lower limbs: *B* = − 11.4; 95% CI − 21.6 to − 1.2; upper limbs: *B* = − 21.4; 95% CI − 36.0 to − 6.8). Having had a treatment in the last 6 months was also predictor of limitations in the time management scale of the WLQ (*B* = 19.2; 95% CI 9.4–29.0).

#### Mental-interpersonal tasks

Patients with a diagnosis of bone sarcoma (*B* = 10.0; 95% CI 1.3–18.7), patients with fibroblastic, myofibroblastic other fibrohistiocytic sarcoma (*B* = 9.9; 95% CI 1.3–18.4) as well as patients with sarcomas classified as “all other” (*B* = 11.2; 95% CI 1.0–21.4) were more limited in their mental and interpersonal tasks in comparison to liposarcoma. A tumor localization on the abdomen/retroperitoneum was also a negative predictor, using this location as reference, all other locations were less affected, again most significantly the upper and lower extremities (thorax: *B* = − 6.2; 95% CI − 16.9 to 4.5; pelvis: *B* = − 4.4; 95% CI − 13.7 to 5.0; lower limbs: *B* = − 7.6; 95% CI − 15.8 to 0.6; upper limbs: *B* = − 16.4; 95% CI − 28.2 to − 4.6; other locations: *B* = − 8.0; 95% CI − 21.0 to 5.0). Having had a treatment on the last 6 months was, again, a significant predictor of limitations in this scale of the WLQ (*B* = 15.4; 95% CI 7.4–23.4). For this group there were no significant differences across sex, age groups or type of employment.

#### Output tasks

Concerning limitations regarding the output tasks, in other words quantity and quality of work, men were significantly less affected than women (*B* = − 6.8; 95% CI − 12.7 to − 1.0). Again, patients > 40 years were significantly more affected than younger patients (40– < 55 years: *B* = 10.1; 95% CI 2.7–17.5; > 55 years: *B* = 9.0; 95% CI 0.6–17.3). Patients with a diagnosis of fibroblastic, myofibroblastic or fibrohistiocytic sarcoma (*B* = 11.2; 95% CI 1.0–21.4), as well as the group of patients classified as “other soft tissue sarcoma” (*B* = 13.2; 95% CI 2.0–24.4) were more limited in the output tasks in comparison to liposarcomas. As in the other scales, using abdominal sarcomas as reference, all other locations were less affected (thorax: *B* = − 10.0; 95% CI − 23.0 to 3.0; pelvis: *B* = − 7.4; 95% CI − 18.6 to 3.7; lower limbs: *B* = − 10.0; 95% CI − 19.7 to − 0.3; upper limbs: *B* = − 19.9; 95% CI − 34.0 to − 5.8; other locations: *B* = − 14.8; 95% CI − 30.1 to 0.5). Having had treatment within the last 6 months was also a significant predictor of limitations in this scale (*B* = 14.9; 95% CI 5.2–24.6).

## Discussion

Among the heterogeneous group of sarcoma patients, we followed-up in the study, more than 20% dropped out of work. At baseline 30% of patients eligible to receive a disability pension and who were employed at time of diagnosis, received this kind of payments. This number cannot be directly compared to the general population statistics, for reference in 2020 a total of 1.8 million (2%) people received a disability pension in Germany (9), while in our study sample of sarcoma patients this was 12.5% (138 of 1101).

Among the analyzed potentially associated variables, we were able to observe several emerging patterns. Thereby it is important to discuss the results not separated from each other. It is plausible to assume, that many of the patients who dropped out of work or receive a disability pension are no longer working precisely because of the acquired limitations after surviving sarcoma. In a sense, drop out of work, receiving a disability pension and burden of work are “competing risks”.

### Socioeconomic factors

The odds of receiving a disability pension increased with age. Likewise, the perceived limitations at the work place increased. These findings are not surprising and have been consistently reported in different cancer entities (Endo et al. 2016; Roelen et al. 2011; Kim et al. 2014; Lima et al. 1997). Women were more burdened in two of the three measured scales of the work limitations questionnaire. There were no significant differences between the genders concerning the odds of dropping out of work and receiving a disability pension. Studies analyzing gender differences in RTW found female sex to be a negative predictor of complete RTW, particularly in hematological cancer (Roelen et al. 2011) or along various types of cancer (Kim et al. 2014; Park et al. 2008).

Self-employed persons were much less likely to drop out of work than the other occupational groups. These results are not easy to interpret due to differences in legal requirements across the different occupational groups. On the one hand, the risk of dismissal does not exist for self-employed persons. It is possible, however, that the economic constraints to which the self-employed are exposed also result from a different approach towards their work than is the case with dependent employees (work ethic) and to more economic pressure to resume their activities as soon as possible after finishing their treatments (Bains et al. 2012). In Germany, self-employed persons usually do not receive any state benefits in the event of occupational disability and must therefore insure themselves. Interestingly, there were no significant differences in limitations at the work place between occupational groups with the exception of white collar workers who had fewer constraints in time management.

### Invariable disease related factors (type, grading, location)

Bone sarcoma patients had a significantly higher burden of work limitations and higher odds of dropping out of their works than liposarcoma patients. Similarly, fibroblastic, myofibroblastic and fibrohistiocytic sarcomas experienced more work limitations than liposarcoma patients. The burden of limitations and analyzed odds were consistently higher in the diverse group of “other soft tissue sarcomas” (comprising synovial sarcomas, angiosarcomas, peripheral nerve sheath tumors and others) which we could not further differentiate due to their rarity. Our results correspond to a certain extent with previous studies of the quality of life of sarcoma patients, that showed the high diversity of sarcoma disease (Eck et al. 2020; McDonough et al. 2019).

We observed differences in the cancer localizations examined. Patients with sarcomas on the abdomen/retroperitoneum in particular had higher odds of receiving an occupational disability pension and were more heavily limited at the work place than patients with sarcomas in the extremities. Even if our results are not directly comparable, the study of Kollar et al. reporting a high RTW of 89% in Swiss patients with sarcoma of the extremities fits in that pattern (Kollár et al. 2021). A probable explanation of the differences may be the prolonged convalescence time after abdominal surgery compared with other localizations.

In agreement with the published literature and not surprisingly, a higher degree of malignancy at diagnosis (grading) was positively associated with receiving a disability pension (Kollár et al. 2021; Coindre et al. 2001).

This observed diversity in invariable disease related factors could be addressed in specific rehabilitation measures. To explore sarcoma diversity in more detail, a linkage of large scale clinical sarcoma databases (Ogura et al. 2017; Jacobs et al. 2015; Trovik et al. 2017) with patient reported outcome data as well as with administrative data would be needed that is not yet established.

### Variable disease factors

The odds of dropping out of working were highest at the beginning of the illness (expiry of the sick leave). Not surprisingly, prevalence of receiving a disability pension increased over time since diagnosis. A positive correlation with limitations at the work place was observed in those who had been treated more recently (in the last 6 months), an observation reflected in the literature (Spelten et al. 2002). This variable could not be included in the models for all three questions due to the time order of data collection. No statistically significant association was found between the odds of dropping out of work over one year and treatment at baseline.

Similarly, there was no association between progressive disease and the odds of dropping out of work; a difficult result to explain was that patients who underwent partial remission were less likely to drop out of work than patients which achieved complete remission. We do not have a straightforward explanation for this observation, though the social security system in Germany may play a role, with patients being able to work at least part time.

The type of received treatment did not had a statistically significant association with limitations at work, but patients who had received a more aggressive treatment (surgery + radiotherapy or surgery + systemic therapy + radiotherapy) had significantly higher odds of dropping out of work over the course of 12 months. This observation, although not surprising, might be a result of the aggressive and sometimes disabling nature of the current therapeutic options (Casali et al. 2018; Strauss et al. 2021). The analysis of treatment options was limited as we could not include complications and toxicity profiles of the specific types of therapy or differentiate treatments further (adjuvant, neoadjuvant). The picture in the literature regarding treatment option is heterogenous. Van Muijen et al. investigated predictors of work ability in different types of cancers (sarcomas not included). They found a negative association with a chemotherapy treatment (Muijen et al. 2017). Bakker et al. reported data on predictors of return to work among survivors of colonic cancer, finding that treatment related factors played an important role in return to work during the first 12 months (Bakker et al. 2020).

### Strengths and limitations

This study is, to our knowledge, the first assessing predictors of drop out of work, disability pension and work limitations among sarcoma patients. The analyses are based on a relatively large data set for this rare disease. The study is probably subject to selection bias. We see this possibility mainly on the level of the study centers. The majority of our patients were recruited in university hospitals and/or specialized centers and might therefore be not representative for all sarcoma patients. Selection bias is also possible at the patient level. Here we suspect a sick survivor bias, as healthy survivors have less frequent contact with our recruiting study centers. Especially patients who are no longer in follow-up care could be reached less easily.

Our results are not directly comparable to studies analyzing RTW. We followed patients over the course of one years who had a job contract or were self-employed at time of study inclusion and recorded drop outs of work. This approach is based on German employment law, according to which patients can be on paid sick leave for a period of 78 weeks without having to terminate their employment. Our approach has the disadvantage that we did not collect data on work limitations among patients who regained an employment contract since study inclusion. Here too, there is the possibility of a selection bias.

With the available data, especially variable disease factors are difficult to analyze as our data collection only took place during a more or less random year of the disease course. Thus, we were not able to integrate variable disease factors in the analysis of research questions 1, as we had no information on when events (receiving a disability pension, receiving treatment) took place.

## Conclusions

Limitations in the work life and predictors of drop out or return to work are increasingly important topics among cancer survivors. To our knowledge, this is the first work reporting predictors of work limitations, disability pension and drop out of work in patients with sarcoma. We were able to identify vulnerable groups reflecting the heterogeneity of the disease. The anatomical and histological variability observed in explored outcomes might be valuable to communicate expectations of the future work life of patients. The results can help to identify groups of patients with higher odds to receive a disability pension over the disease curse, such as older patients, those with rarer sarcoma histological types or patients with abdominal or retroperitoneal located sarcoma. The results also emphasize the need for better structures for the reintegration of patients in their work environment.

## Supplementary Information

Below is the link to the electronic supplementary material.

Supplementary file1 (DOCX 19 KB)

## Data Availability

The datasets generated during and analyzed during the current study are available from the corresponding author on reasonable request.
