# Supplementary material for: Working situation and burden of work limitations in sarcoma patients: results from the multi-center prospective PROSa study
Source: J Cancer Res Clin Oncol. 2023 Jan 10;149(9):6009–21. doi: 10.1007/s00432-022-04556-3 (PMC10356622; doi:10.1007/s00432-022-04556-3)
Supplement: Supplementary file 1 — Supplementary file1 (DOCX 19 KB) [file 432_2022_4556_MOESM1_ESM.docx]

**Online Appendix. Baseline Description. Model Variables Disability Pension. Frequencies.**

| **Variable**  **-value** | **Employment at baseline**  **N= 254 (69.8%)**  **N (%)** | **Disability pension at baseline N=110 (30.2%)  N (%)** | **all  N=364 N (column %)** |
| --- | --- | --- | --- |
| Sex |  |  |  |
| -female | 125 (65.4) | 66 (34.6) | 191 (52.5) |
| -male | 129 (74.6) | 44 (25.4) | 173 (47.5) |
| Age at study inclusion |  |  |  |
| -18-<40 years | 59 (88.1) | 8 (11.9) | 67 (18.4) |
| -40-<55 years | 104 (67.1) | 51 (32.9) | 155 (42.6) |
| -55 years and older | 86 (63.2) | 50 (36.8) | 136 (39.0) |
| School education |  |  |  |
| -Secondary school (8/ 9 years) | 32 (58.2) | 23 (41.8) | 55 (15.1) |
| -Secondary school (10 years) | 86 (60.6) | 56 (39.4) | 142 (142) |
| -(Vocational) baccalaureate/ high school | 131 (83.4) | 26 (16.6) | 157 (43.1) |
| -other | 5 (50.0) | 5 (50.0) | 10 (2.7) |
| Occupational status |  |  |  |
| -Blue collar worker | 42 (60.0) | 28 (40.0) | 70 (19.2) |
| -White collar worker | 176 (71.3) | 71 (28.7) | 247 (67.9) |
| -Self employed | 29 (87.9) | 4 (12.1) | 33 (9.1) |
| -Not applicable/ unknown | 7 (50.0) | 7 (50.0) | 14 (3.8) |
| Time since diagnosis t0 |  |  |  |
| -1-<2 years | 70 (83.3) | 13 (16.7) | 84 (23.1) |
| -2-<5 years | 92 (69.7) | 40 (30.3) | 132 (36.3) |
| ->5 years | 92 (62.2) | 56 (37.8) | 148 (40.7) |
| Sarcoma Type |  |  |  |
| -liposarcoma | 52 (70.3) | 22 (29.7) | 74 (20.3) |
| -bone sarcoma | 58 (79.5) | 15 (20.5) | 73 (20.1) |
| -GIST | 25 (65.8) | 13 (34.2) | 38 (10.4) |
| -unclassified sarcoma | 27 (75.0) | 9 (25.0) | 36 (9.9) |
| -fibroblastic, myofibroblastic, fibrohistiocytic sarcoma | 35 (74.5) | 12 (25.5) | 47 (12.9) |
| -leiomyosarcoma | 23 (52.3) | 21 (47.7) | 44 (12.1) |
| -other soft tissue sarcomas | 34 (65.4) | 18 (34.6) | 52 (14.3) |
| Site |  |  |  |
| -abdomen/ retroperitoneum | 53 (55.2) | 43 (44.8) | 96 (26.4) |
| -thorax | 32 (77.8) | 6 (22.2) | 27 (7.4) |
| -pelvis | 37 (67.3) | 18 (32.7) | 55 (15.1) |
| -lower limbs | 113 (79.6) | 29 (20.4) | 142 (39.0) |
| -upper limbs | 21 (84.0) | 4 (16.0) | 25 (6.9) |
| -other | 9 (47.4) | 10 (52.6) | 19 (5.2) |
| Grading at diagnose |  |  |  |
| -low grade | 49 (84.5) | 9 (15.5) | 58 (15.9) |
| -high grade | 115 (65.0) | 62 (35.0) | 177 (48.6) |
| -not applicable | 72 (72.7) | 27 (27.3) | 99 (27.2) |
| -unknown | 18 (60.0) | 12 (40.0) | 30 (8.2) |

Abbreviations. GIST: gastrointestinal stromal tumour.
